# Wheelchair Skills Test Outcomes across Multiple Wheelchair Skills Training Bootcamp Cohorts

**DOI:** 10.3390/ijerph19010021

**Published:** 2021-12-21

**Authors:** Edward Giesbrecht

**Affiliations:** College of Rehabilitation Sciences, University of Manitoba, R106-771 McDermot Avenue, Winnipeg, MB R3E OT6, Canada; ed.giesbrecht@umanitoba.ca; Tel.: +1-204-977-5630

**Keywords:** mobility, assistive technology, social participation, community integration, wheelchair, rehabilitation, graduate education

## Abstract

User training is a critical component of wheelchair service delivery to ensure individuals with a mobility impairment can negotiate environmental barriers and promote their social participation. A wheelchair “bootcamp”, delivered during professional preparation education, is one strategy to better prepare occupational therapists for clinical rehabilitation practice by developing their own wheelchair skills. The purpose of this study was a retrospective review of a large dataset of student cohorts from a single site and delineate bootcamp effects on the Wheelchair Skills Test-Questionnaire (WST-Q) scores. Participant data from eight cohorts was consolidated (*n* = 307). Comparison of two WST-Q scoring formats revealed significantly lower scores for cohorts using the 4-point version, which was subsequently standardized to the other 3-point version. WST-Q change scores were similar between cohorts, and differences were more reflective of variability in skill level prior to bootcamp than post-bootcamp scores. Students were able to master most basic and intermediate level skills, while advanced skill acquisition was much more variable. This study provides more precise point estimates of wheelchair skill acquisition among occupational therapy students than previous studies. While confirming the benefits of bootcamp education, recommendations for further investigation were identified.

## 1. Introduction

Best practice in wheelchair service provision should entail user and caregiver training in mobility skills [1,2]. Furthermore, the use of a structured evidence-informed training program, such as the Wheelchair Skills Program (WSP) [3], has demonstrated significant and clinically important improvements to the skill capacity and performance of wheelchair users [4,5], which in turn can positively impact participation, wheelchair self-efficacy, and quality of life [6,7]. While such evidence is persuasive, delivery of skills training in clinical practice remains variable [8] and is often brief and focused on the most basic wheelchair skill set [9]. In addition to insufficient time and resources, service providers frequently identify a lack of confidence and competence to teach wheelchair skills [10]. One strategy to address this issue is to provide WSP training to clinicians during their professional education, prior to entering practice. Specifically, intensive experiential learning workshops (i.e., wheelchair “bootcamps”) have been demonstrated to produce substantive improvements in occupational therapy students’ capacity and self-efficacy regarding wheelchair use [10,11,12], and their self-efficacy to deliver such training to their future clients [12,13]. While wheelchair bootcamps are an expeditious pedagogy within a congested professional education program, incorporating motor learning principles inherent in the WSP is challenging. 

To evaluate training interventions (whether among wheelchair users or those delivering skill training), some measure of skill capacity (can one perform a skill) or performance (does one perform the skill in a life context) is imperative. The most frequently reported outcome in the literature is the Wheelchair Skills Test (WST): an associated component of the WSP [3]. The psychometric properties and evaluation of the WST have been previously reported in multiple publications and have been used extensively for both research and clinical applications [14]. The WST is an objective evaluation of skill capacity administered and scored by a clinician or trainer, with variations for different target populations (i.e., device user or caregiver) and wheelchair device types (i.e., manual or power/scooter). The WST is also available in a questionnaire version (WST-Q) where the device user conducts a self-evaluation of the same set of skills [15,16]. WST-Q scores are highly correlated with the objective test version, although WST-Q scores tend to be slightly higher [16]. 

The WSP includes a comprehensive manual outlining how to teach wheelchair skills (e.g., motor-learning principles, providing feedback) and what to teach (e.g., detailed description of strategies to perform each specific skill). The WSP (and its associated WST/WST-Q measures) have evolved since the initial format (WST version 1.0) was validated in 2002 (14) and became widely available through the host website [3], with successive versions (WST 2.4, 3.2, 4.1, 4.2, 4.3, 5.0, 5.1 and 5.2) being updated periodically. The original WST measure included 33 individual skills, each scored on a 3-point ordinal scale providing a composite percentage score. In addition, three subscale scores can be extracted: indoor (i.e., basic), community (i.e., managing more difficult barriers typically encountered outside), and advanced (i.e., highly challenging) skills. Changes in each subsequent version of the measure have generally been minor, such as modifications to individual item wording, consolidation/separation of skills, and the addition/subtraction of items. A more substantive change occurred with version 5.0 (released in 2018), where the scoring scheme for each skill item query (“Can you do it”) was modified from a 3-point scale (0–2) to a 4-point scale (0–3). In subsequent versions (5.1 released in 2020), this adaptation was retained with the WST while the WST-Q reverted to a 3-point scale. Publications on wheelchair skill training using different versions of the test exist, but there are currently few studies that compare versions or any potential impact on scores and the interpretation of findings.

Consequently, while studies evaluating the impact of wheelchair bootcamps on rehabilitation students report significant improvements in skill capacity from the WST-Q, the results can vary considerably among different cohorts. This variability may be due to differences between cohorts, such as baseline skill capacity and self-efficacy, delivery of the bootcamp intervention, and use of different WST-Q versions. Furthermore, the relatively small size of individual study cohorts provides lower precision of point estimates with wide confidence intervals and makes the analysis of individual and skill subsets difficult. A larger database was available with WST-Q outcomes for eight successive cohorts of occupational therapy students attending a wheelchair skills bootcamp as part of their professional education. Therefore, the purpose of this study was to explore the effect of a training bootcamp on wheelchair skills outcomes among multiple cohorts at a single site. The specific objectives were to identify: (a) any effect different WST-Q version scoring scales might have on participant scores; (b) the effect on WST-Q scores across and between cohorts; (c) how outcomes vary according to subscales and individual skills; and (d) the relationship between baseline competency and skill acquisition.

## 2. Materials and Methods

**Figure 1:**
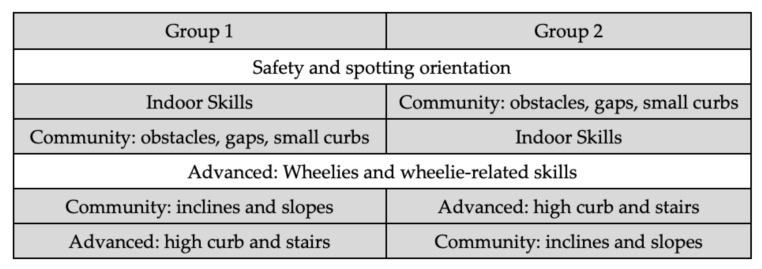
Typical sequence of WST-Q skills training in the bootcamp. Note: WST-Q = Wheelchair Skills Test Questionnaire.

This study was a retrospective review of eight cohorts within a single-site occupational therapy university program. Students had already received training in assessment, prescription, fitting, and configuration of manual wheelchairs. The bootcamps were conducted annually between 2013 and 2020 for students in their final year. They were 4–4.5 h in duration with several groups of approximately 15–20 students each, conducted indoors over a weekend using a variety of lightweight and ultralightweight wheelchairs. The typical sequence of skills training is provided in Figure 1; more specifics about bootcamp administration were reported previously [10,13]. Between 2013 and 2015, bootcamps were conducted as extracurricular workshops where students voluntarily enrolled, while those from 2016 onward were incorporated into the core curriculum. All bootcamp participants were asked to complete the WST-Q in the week preceding (pre-bootcamp) and then again immediately following completion (post-bootcamp). To support best practice, updated versions of the WST-Q were incorporated as they became available. Completion of the questionnaire was not compulsory, nor did it impact students’ evaluation in the program. After the bootcamp, questionnaire responses were transcribed into an electronic spreadsheet. All data for each cohort were anonymized prior to the retrospective review. To address the four different versions of the WST-Q used (2013–2014 v 4.2; 2015–2017 v 4.3; 2018–2019 v 5.0; 2020 v 5.1), a comprehensive inventory of all 34 possible questionnaire items from the four different versions was created within the spreadsheet. Then, for each cohort, only responses to the skill items present in their WST-Q version were inserted. WST-Q scores could then be calculated for each respondent based on the WST-Q version used, and by tabulating individual item scores across different WST-Q versions and cohorts. Following ethical approval from the University of Manitoba, cohort data were consolidated into a single database where participants were assigned a unique ID number and one additional variable (cohort year) was added.

### Data Analysis

Some respondents inadvertently did not answer one or more items in the WST-Q questionnaire. There were 45 responses missing out of a total 20,090 data points, spread across 26 different items with no single question having more than two non-responses. Given this small proportion (0.2%) and the likelihood that data were missing at random, imputed values were inserted to complete the dataset. Since individual WST-Q items are scored ordinally, the imputed value used was the median of all responses to that item within the same cohort. 

To address the objectives regarding the effect of WST-Q version and between-cohort differences, scores were compared using Analysis of Variance (ANOVA) with Gabriel post-hoc analyses for homogenous subgroups. Where equal variance could not be assumed, the Welch test was used with Games–Howell post-hoc analysis. Descriptive statistics and point estimates (mean and 95% CI) were used to identify WST-Q scores for the entire sample and for individual cohorts for the second objective. For objective three, WST-Q subscales (indoor, community, and advanced) and individual item scores were also evaluated using descriptive statistics. Finally, the fourth objective examining relationships between baseline and change score was evaluated using Pearson correlation.

## 3. Results

As the data were anonymized with no personal identifiers included, there are no summary demographics. The eight cohorts reflect the general demographics of students within this university program over the same time period (age = 25.3 ± 4.6 years; 88.3% female).

### 3.1. Effect of WST-Q Version on Scores

ANOVA results for pre- (F = 11.4; df = 7, 299, *p* ≤ 0.001) and post-bootcamp scores (F = 30.0; df = 7, 299, *p* ≤ 0.001) across cohorts were statistically significant, with post-hoc analyses indicating cohort 6, 7, and 8 mean scores were lower than those of cohorts 1–5. ANOVA for change score also exhibited a statistically significant difference (F = 5.2; df = 7, 299, *p* ≤ 0.001), with post-hoc analysis indicating cohorts 6 and 7 were lower than the remaining homogenous subset. As follow-up, ANOVA with 6 and 7 removed did not find a significant difference among the remaining cohorts (F = 2.1; df = 5, 212, *p* = 0.06). 

**Figure 2:**
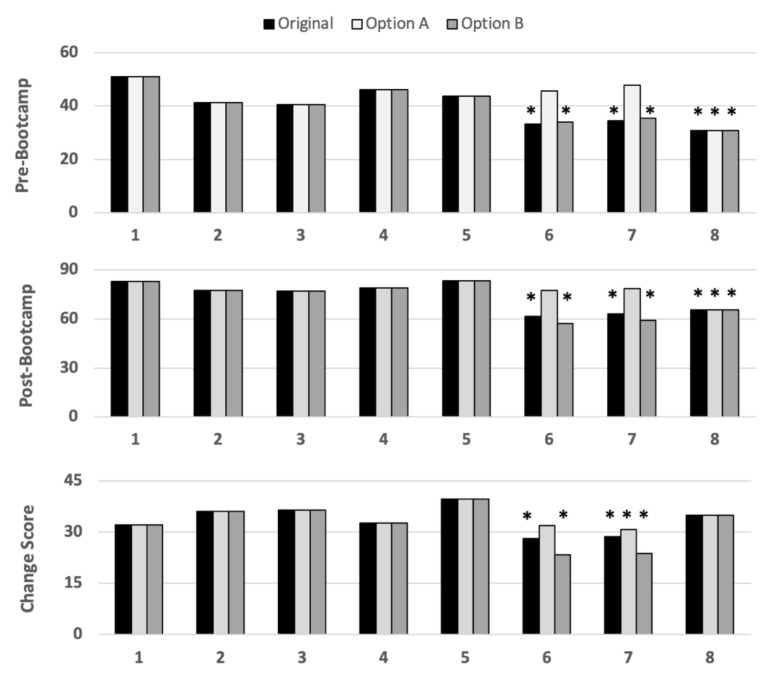
Subplots of mean scores by cohort using original and alternative WST-Q scoring. Note: asterisks indicate cohorts that were significantly lower than the remaining cohorts (*p* < 0.05) when using the original (black), option A (white), and option B (grey) scoring scales; WST-Q = Wheelchair Skills Test Questionnaire.

As only cohorts 6 and 7 employed a 4-point scale, their scoring was adapted to reflect a 3-point scale as per the remaining cohorts. To be comprehensive, two alternatives were used. Both retained the lowest “no” response as “0”. Option A conflated “very well” and “yes” as “2”, while option B conflated “yes” and “partially” responses as “1”. ANOVA was repeated with option A and only cohort 8 remained significantly lower for both pre (F = 8.4; df = 7, 299; *p* < 0.001) and post (F = 14.8; df = 7, 299; *p* ≤ 0.001) scores and only cohort 7 had a significantly lower change score (F = 2.8; df = 7, 299; *p* < 0.008). With option B, comparable statistically significant cohort differences remained as per the original data (values not reported) (See Figure 2). Consequently, the revised 3-point scale data (using option A) was retained for cohorts 6 and 7 for all remaining analyses.

### 3.2. Effect of Bootcamp Training: Point Estimates

Across all participants (*n* = 307), mean WST-Q scores (95% CI) were 43.5% (41.9; 45.2) for pre-bootcamp, 77.5% (76.3; 78.8) for post-bootcamp, and 34.3% (32.9; 35.7) for change score (i.e., improvement). Mean WST-Q scores for individual cohorts are presented in Table 1.

### 3.3. Effect of Bootcamp Training: Skill Complexity (Subscales) and Individual Skills

Mean scores for WST-Q subscale scores across all participants are provided in Table 2. 

### 3.4. Effect of Bootcamp Training: Relationship between Baseline and Change Score

Individual item mean scores and rankings are reported in Table 3. On the indoor subscale, no item had less than 95% of participants scoring at least 1 (“*Yes, with difficulty*”) post-bootcamp and only two had less than 80% scoring 2 (“Yes”): *level transfers* (76%) and *turning backwards* (69%). On the community subscale, only the *ground to wheelchair transfer* item had less than 95% of participants scoring at least 1 post-bootcamp and four items had less than 80% of participants scoring 2: *moving down* (76%) *or up* (73%) *a steep incline*; *folding/unfolding wheelchair* (56%); and *ground to wheelchair transfer* (49%). On the advanced subscale, the *wheelie for 30 s* item had the highest proficiency with 78% of participants scoring at least a 1 and 29% scoring 2, followed by *descending stairs with a railing* where 73% scored at least 1 and 36% scoring 2. Four additional items saw at least 40% of participants scoring 1 or more: *getting down* (66%) *or up* (55%) *a 6″ curb* and *moving forward/backward* (48%) or *turning* (44%) *in a wheelie position*. The remaining skills of *descending a 6″ curb* (27%) or *steep ramp* (24%) *in a wheelie position* and *ascending stairs* (10%) had a low prevalence of participants scoring at least 1.

## 4. Discussion

The WSP and the associated WST-Q outcome measures have gone through a variety of revisions since first publication, demonstrating a dynamic response to evolving evidence in the field. The expansion of the WST-Q scoring scale, from three to four response points in version 5.0, intended to increase measurement granularity in order to inform clinical practice (e.g., to identify smaller improvements that might not meet the threshold of the mid-point). While this could potentially capture incremental improvement, in the two cohorts where this scale was used, the magnitude of both pre- and post-bootcamp scores was significantly lower than all but one cohort, and the resultant change score was significantly lower than all other cohorts. One argument might be that the 4-point scale is successful in addressing a ceiling effect; however, even the most proficient cohort in this study did not exceed a mean score of 84%. Alternately, it might be the case that cohorts 6 and 7 (who used the 4-point scale) might have actually begun with a much lower level of proficiency than the first five cohorts, as was also the case with cohort 8. However, the change score in cohorts 6 and 7 was significantly smaller than all of the remaining cohorts (including cohort 8). Given the high (negative) correlation between pre- and change-score, cohorts 6 and 7 would be expected to show larger change scores, as was the case with cohort 8. Furthermore, after adjusting individual scores to a 3-point scale in cohorts 6 and 7, anomalies relative to other cohorts were largely eliminated. 

The 4-point scoring scale may well serve an intended purpose for clinical practice and this is worthy of further study. The objective (observer scored *capacity*) WST uses this scale, while the most recent (self-reported *performance*) WST-Q version has reverted to a 3-point scale. Use of the 4-point scale WST-Q for research purposes, particularly where scores are compared with the existing literature, may warrant some forethought. In our application with occupational therapy students, the 4-point scale seems to reduce estimates of pre- and post-bootcamp score and improvement. However, we cannot generalize such findings to interventions with wheelchair users nor to applications with the objective WST, and this should be considered in future investigation.

This study was successful in obtaining more precise estimates of participant scores than have previously been obtained among occupational therapy students following their wheelchair bootcamp experience. After standardizing WST-Q scores to the 3-point scale, there was relative uniformity in the rate of improvement, between roughly 31% and 39%. Across all cohorts, participants experienced a mean improvement of 34.3%. This is lower than previously published bootcamp studies reporting improvements of 36.5% to 47.2% [10,11,12,13], although previous studies have much wider confidence intervals. Several factors might contribute to this finding. First, individuals and cohorts vary in their responsiveness to improving skill capacity, as demonstrated by the range of change score found between cohorts in this study. Second, cohorts with lower baseline skill capacity are likely to demonstrate larger improvements post-bootcamp, evidenced by the high correlation reported in our results. Single cohorts of smaller size may tend to overestimate the true intervention effect, whereas in this dataset, most cohorts included responses from nearly all students enrolled, providing considerable variability in skill proficiency. Furthermore, change score, while useful, may not capture a full explanation of bootcamp benefits. Our post-bootcamp mean score of 77.5% is comparable to other smaller studies that range from 78.7% to 83.4% [10,11,12,13]. Cohort scores were very comparable in our study aside from cohort 8, which demonstrated atypically low pre-bootcamp scores. We cannot be certain why this particular cohort expressed substantially lower baseline capacity but it is likely that, as the first cohort to experience COVID pandemic-related changes in their professional education program (i.e., largely virtual learning and fieldwork; restrictions during the bootcamp) [17,18], these students had less opportunity to acquire experience or self-efficacy with wheelchair use. Broadly speaking, occupational therapy students have entered the bootcamp with more diverse levels of skill while finishing the bootcamp at relatively similar capacity (typically close to 80%), with larger change scores typically correlating to a lower baseline score. 

Collectively, participants tended to approach proficiency on both the indoor and community subscales following the bootcamp. These subscales were strongly correlated with baseline scores, suggesting those with lower incoming capacity were likely to see larger improvement. This is intuitive as lower scores offer more room for improvement whereas higher scores can create a ceiling effect. Scoring on the advanced subscale was considerably different. Participants almost universally began at a very low capacity (less than 5%), which likely explains the negligible correlation with change score. It is conceivable that students who entered the bootcamp as more proficient with indoor and community subscale skills were able to focus more attention on the advanced skills; however, this association proved to be minimal. Alternately, students who acquire more foundational skills might likewise acquire more advanced skills during training, but this association was only slightly larger. The factors that contribute this variability in learning require further investigation. While it is evident most participants were able to make substantive improvements with the most challenging skills, the distribution skewed towards the low end. 

Several factors might presumably be responsible for this variability. While more than 75% of participants reported successfully performing the stationary wheelie skill, applying that skill in other contexts (e.g., maneuvering forward, backward and turning; descending a high curb or steep ramp) is more difficult. This is not entirely surprising as participants first need to learn how to perform skills in a stable position (horizontal orientation) before being able to integrate the wheelie skill elsewhere [19,20]. A number of factors likely contribute to this. First, the duration of the boot camp is not sufficient for many participants to make this progression, particularly if they begin at a lower capacity. Second, while participants observe skill demonstrations, they are not compelled to perform or practice skills for which they feel unready or unsafe. Consequently, some students do not attempt “higher risk” skills like ascending/descending a 6″ curb and might benefit from “follow-up” training opportunities with more individualized attention. Finally, motor learning theory suggests that individuals require time to reflect on and process individual skill performance before trying to integrate multiple skills components, and that considerable repetition in practice is required to become proficient with new motor skills [21,22]. While pragmatic, the boot camp format is not optimally configured for motor learning, particularly for those with lower entrance capacity scores (i.e., previous experience). However, given that participants have shown considerable success in learning individual skills (particularly indoor and community skills), there is optimism that through the remainder of their professional education (academic and fieldwork) they have additional opportunity to integrate and combine these skills as they move into practice. Previous publication around general retention of skills and self-efficacy for skill performance and clinical application affirm this potential [13]. Integrating the WSP into occupational therapy students’ professional education using a bootcamp format should therefore have benefits for future clinical practice, increasing the likelihood that graduates will integrate skills training as part of wheelchair service provision [2]. Furthermore, their capacity to demonstrate advanced skills may lead to a more comprehensive scope of skill training with future patients, which is currently an infrequent practice [8,9].

Some issues should be acknowledged in interpreting these findings. Transformation of the raw data from two cohorts better reflects a standardized scoring approach to the WST-Q and comparison with published literature but is a modification to participants’ original questionnaire responses. The WST/WST-Q employs an ordinal scoring scale for clinical expediency, but the potential uncertainty between scoring intervals may impact responsiveness of these measures [23]. The results reflect the effects of WSP training for occupational therapy students, but caution should be used in generalizing findings to other populations, particularly authentic wheelchair users. This study represents the most comprehensive evaluation to date of skill acquisition following wheelchair bootcamp training and highlights a number of avenues for future study. A more in-depth evaluation of factors impacting variability in improvement with advanced skills could inform improvements in the organization and delivery of the skills bootcamp, and application to clinical populations. Objective testing of students’ skill capacity using the WST (in addition to performance self-reporting using the WST-Q) might provide additional insight into bootcamp training and might further elucidate how the different scoring scales reflect evaluation using the WST. Prospective evaluation of professional education (e.g., with and without bootcamp training; extent of post-bootcamp skill and self-efficacy) might help elucidate the degree to which clinician factors, versus environmental factors, are barriers or facilitators to future clinical practice.

## 5. Conclusions

Evaluation of a large dataset comprising eight successive bootcamp cohorts afforded greater precision in estimating the effect of training on occupational therapy students’ wheelchair skills. The 3-point scoring scale on the WST-Q measure provided greater comparability between cohorts. Individual student and mean cohort scores appear to vary more at baseline, with post-bootcamp scores typically rising to about 78%. Consequently, higher reported change scores may be more reflective of low baseline skill than necessarily more effective training. Bootcamp participants became quite proficient with foundational and intermediate level skills, whereas acquisition and mastery of advanced skills was variable and is not strongly associated with indoor and community level skills. Further investigation of the factors that facilitate this learning could enhance delivery of bootcamp training and the preparation of occupational therapists for clinical practice.

## Figures and Tables

**Table 1:** WST-Q mean % scores (95% CI) by cohort.

Cohort	*n*	Version	Pre Score	Post Score	Change Score
1	36	4.2	51.1 (46.1; 56.1)	83.1 (80.0; 86.3)	32.0 (28.2; 35.8)
2	14	4.2	41.2 (34.7; 47.8)	77.3 (73.8; 80.8)	36.0 (30.0; 42.1)
3	31	4.2	40.5 (35.3; 45.7)	77.0 (74.5; 79.4)	36.5 (32.4; 40.6)
4	45	4.2	46.3 (42.1; 50.4)	79.0 (76.1; 81.8)	32.7 (29.3; 36.1)
5	42	4.2	43.8 (40.2; 47.3)	83.4 (81.2; 85.7)	39.7 (36.1; 43.2)
6	46	5.0	33.3 (29.4; 37.3)	61.4 (58.3; 64.5)	28.1 (25.8; 30.4)
6 ^1^			45.6 (41.0; 50.1)	77.4 (74.4; 80.5)	31.9 (28.5; 35.2)
7	48	5.0	34.6 (31.3; 37.9)	63.3 (60.3; 66.3)	28.7 (25.8; 31.6)
7 ^1^			47.9 (44.1; 51.8)	78.6 (75.9; 81.3)	30.7 (27.4; 33.9)
8	45	5.1	30.8 (26.5; 35.0)	65.7 (62.1; 69.3)	34.9 (31.5; 38.3)

^1^ Scores adjusted from a 0–3 to 0–2 scoring scale. Note: WST-Q = Wheelchair Skills Test Questionnaire.

**Table 2:** WST-Q subscale mean % score (95% CI) and correlation between pre and change score ^1^.

Scale	Pre Score	Post Score	Change Score	Pearson	*p*	R^2^
Indoor	67.9 (65.9; 69.9)	94.8 (93.9; 95.9)	26.9 (25.2; 28.6)	−0.893	<0.001	0.80
Community	47.7 (45.3; 50.0)	89.2 (88.0; 90.4)	41.5 (39.6; 43.4)	−0.862	<0.001	0.74
Advanced	4.2 (3.3; 5.2)	34.7 (32.0; 37.4)	30.5 (27.9; 33.0)	0.013	0.822	<0.01
Total WST-Q	43.6 (41.9; 45.2)	77.5 (76.3; 78.8)	34.3 (32.9; 35.7)	−0.701	<0.001	0.49

^1^ Scores reflect 0–2 scoring scale; WST-Q = Wheelchair Skills Test Questionnaire.

**Table 3:** WST-Q individual item mean scores (out of 2) ^1^ and ranking (*n* = 307) ^2^.

	Skill Item ^3^	Mean Item Score ^4^		
		Pre	Post	Change
Indoor Subscale	Moving straight forwards	1.94	2.00	0.06
	Moving straight backwards	1.77	1.98	0.21
	Turning a corner forwards	1.62	1.97	0.36
	Turning a corner backwards (*n* = 262)	0.83	1.66	0.82
	Turning around in a small space	1.37	1.96	0.59
	Moving the wheelchair sideways	0.89	1.86	0.97
	Open and go through a hinged door	0.94	1.84	0.90
	Reach up for something overhead (*n* = 168)	1.31	1.97	0.66
	Pick up a small object	1.19	1.93	0.74
	Removing weight from buttocks	1.71	1.97	0.27
	Transferring at the same height	1.30	1.73	0.43
	Folding/opening wheelchair	1.03	1.48	0.45
	Moving forwards a longer distance	1.66	1.96	0.30
	Avoiding people/sudden stop (*n* = 262)	1.33	1.87	0.53
Community Subscale	Moving up a slight incline	1.37	1.92	0.55
	Moving down a slight incline	1.49	1.94	0.45
	Moving up a steep incline	0.64	1.72	1.08
	Moving down a steep incline	0.72	1.76	1.05
	Moving across a side-slope	1.07	1.93	0.86
	Moving across a soft surface	0.94	1.82	0.88
	Getting over a gap	0.43	1.81	1.38
	Getting over an obstacle	0.86	1.89	1.02
	Getting up a 2″ curb	0.59	1.81	1.22
	Getting down a 2″ curb	0.74	1.81	1.07
	Getting from ground to wheelchair	0.56	1.29	0.73
Advanced Subscale	Getting up a 6″ curb	0.16	0.76	0.60
	Getting down a 6″ curb	0.24	0.93	0.69
	Doing a wheelie for 30 s	0.12	1.07	0.95
	Turning around while in a wheelie	0.03	0.58	0.55
	Down a steep ramp in a wheelie	0.01	0.30	0.29
	Down a 6″ curb in a wheelie	0.02	0.36	0.34
	Down a flight of stairs with railing	0.04	1.09	1.06
	Up a flight of stairs with railing (*n* = 139)	0.06	0.12	0.06
	Forward and backward in a wheelie (*n* = 139)	0.07	0.60	0.53

^1^ Scores reflect 0–2 scoring scale; ^2^ items that appear in only some WST-Q versions include the *n* value next to the description; ^3^ item wording has been simplified for readability; ^4^ item scores are ranked from highest (dark grey) to lowest (white) in each column. Note: WST-Q = Wheelchair Skills Test Questionnaire.

## Data Availability

The data presented in this study are available on request from the corresponding author. The data are not publicly available due to departmental requirements.
